# Fluorinated Galactoses Inhibit Galactose-1-Phosphate Uridyltransferase and Metabolically Induce Galactosemia-like Phenotypes in HEK-293 Cells

**DOI:** 10.3390/cells9030607

**Published:** 2020-03-03

**Authors:** Verena Janes, Simona Grabany, Julien Delbrouck, Stephane P. Vincent, Johannes Gottschalk, Lothar Elling, Franz-Georg Hanisch

**Affiliations:** 1 Institute of Biochemistry II, Medical Faculty, University of Cologne, 50931 Koeln, Germany; verena.janes@rwth-aachen.de (V.J.); sgrabany@smail.uni-koeln.de (S.G.); 2 Department of Chemistry, Laboratory of Bio-organic Chemistry, University of Namur (FUNDP), B-5000 Namur, Belgium; julien.delbrouck@unamur.be (J.D.); stephane.vincent@unamur.be (S.P.V.); 3 Helmholtz Institute for Biomedical Engineering, 52074 Aachen, Germany; j.gottschalk@biotech.rwth-aachen.de (J.G.); l.elling@biotech.rwth-aachen.de (L.E.)

**Keywords:** classic galactosemia, galactose-1-phosphate uridyltransferase (GALT), glycosylation, glycoprotein, membrane rafts

## Abstract

Genetic defects of human galactose-1-phosphate uridyltransferase (*h*GALT) and the partial loss of enzyme function result in an altered galactose metabolism with serious long-term developmental impairment of organs in classic galactosemia patients. In search for cellular pathomechanisms induced by the stressor galactose, we looked for ways to induce metabolically a galactosemia-like phenotype by *h*GALT inhibition in HEK293 cells. In kinetic studies, we provide evidence for 2-fluorinated galactose-1-phosphate (F-Gal-1-P) to competitively inhibit recombinant *h*GALT with a K_I_ of 0.9 mM. Contrasting with hepatic cells, no alterations of N-glycoprofiles in MIG (metabolic induction of galactosemia)-HEK293 cells were revealed for an inducible secretory netrin-1 probe by MALDI-MS. Differential fluorescence-activated cell sorting demonstrated reduced surface expression of N-glycosylated CD109, EGFR, DPP4, and r*h*MUC1. Membrane raft proteomes exhibited dramatic alterations pointing to an affection of the unfolded protein response, and of targeted protein traffick. Most prominent, a negative regulation of oxidative stress was revealed presumably as a response to a NADPH pool depletion during reduction of Gal/F-Gal. Cellular perturbations induced by fluorinated galactoses in normal epithelial cells resemble proteomic changes revealed for galactosemic fibroblasts. In conclusion, the metabolic induction of galactosemia-like phenotypes in healthy epithelial/neuronal cells could support studies on the molecular pathomechanisms in classic galactosemia, in particular under conditions of low galactose stress and residual GALT activity.

## 1. Introduction

Galactose-1-phosphate uridyltransferase (GALT; EC 2.7.7.12), as one of the enzymes in the Leloir pathway, is responsible for the conversion of galactose-1-phosphate to UDP-galactose. A GALT deficiency (classic galactosemia, refer to entry #5056 in the Disease Database), which leads to an accumulation of galactose-1-phosphate, is associated with a potentially lethal acute hepatotoxic syndrome, unless affected newborns are kept under a galactose-restricted diet. Endogenously synthesized galactose however, can exert chronic cell toxic effects and worsen the prospects of patients on longer terms. Most patients suffer from a cognitive impairment, from speech defects, motor function disturbances, and a hypergonadotropic hypogonadism in female patients [1].

Besides direct cell-toxic effects exerted by accumulated galactose-1-phosphate, an indirect effect on the glycosylation machinery mediated by reduced provision of the substrate UDP-Gal could lead to altered protein glycosylation with impact on cellular functions in general and on the expression of membrane glycoproteins in particular. Dys-glycosylations of secreted proteins, like transferrin and glycopeptide hormones, were reported [2], however no reports are currently available for aberrant N-glycosylation of membrane glycoproteins in classic galactosemia. Our own studies on membrane-bound N-glycoproteins from patient-derived urinary exosomes had indicated altered N-glycosylation [3], however this finding was later related to absorbed serum glycoproteins having passed the renal filter due to a subclinical kidney insufficiency [4]. A differential proteomic study in a fibroblast model demonstrated that a series of membrane receptors, which mostly belong to the N-linked glycoproteins, showed diminished expression [5]. Besides CD109, DPP4, and others, EGFR is highlighted, because the receptor was previously reported to show decreased membrane expression in GALT-deficient fibroblasts grown under galactose stress [6]. These findings are in agreement with known functions of glycans located in the stem region of the transmembrane proteins, i.e., their involvement in sorting for apical targeting of N-glycoproteins [7]. However, they could be also explained by altered gene expression.

Observed effects of galactose stress on membrane proteomes of galactosemic cells could be causative for perturbations in signaling cascades and could give hints to affected pathways and the molecular pathomechanisms of the disease. However, cell models that are currently available restrict studies of differential protein expression on patient-derived lymphoblasts and fibroblasts. It would be important for further understanding of molecular pathomechanisms underlying classic galactosemia to study epithelial, and in particular, neuronal cells, as developmental impairment of these cells could be causative for the observed mental syndromes. In particular, neuroepithelial cells (also called neuroblasts), which are regarded as “stem cells” of the nervous system, could be of importance, as these cells differentiate further into neurons, astrocytes, and other glial cells. They develop during embryogenesis with formation of the neural tube, but also in adult neurogenesis restricted to specific areas of the brain.

Here, we report on the attempt to induce in HEK293 cells, which exhibit an epithelial/neuronal phenotype [8], a classic galactosemia-like phenotype by following a metabolic approach based on the partial inhibition of *h*GALT with 2-fluorinated galactose derivatives, called metabolic induction of galactosemia-like phenotype (MIG). To increase cellular responses to low stressor concentrations, galactose was kept in most experiments at equal molarity to glucose (0.1%, low-Glc/low-Gal medium).

## 2. Materials and Methods

### 2.1. Fluorinated Galactose Derivatives and Other Materials

2-Fluoro-2-deoxy-galactose (F-Gal, Toronto Research Chemicals, North York, Canada) was purchased from BIOZOL, Eching, Germany. 2-Fluoro-2-deoxy-galactose-1-phosphate (F-Gal-1-P) was prepared by enzymatic phosphorylation using recombinant galactose kinase 1 (see below). 2,2′-Difluoro-galactose (F_2_-Gal), 2,2′-difluoro-galactose-1-phosphate (F_2_-Gal-1-P), and per-O-acetyl-2,2′-difluoro-galactose-1-phosphate (O-acetyl-F_2_-Gal-1-P) were chemically synthesized (see below). UDP-glucose (UDP-Glc) and glucose-1-phosphate (Glc-1-P) were purchased from Sigma Aldrich (Munich, Germany).

### 2.2. Preparation of 2-fluoro-2-deoxy-galactose-1-phosphate (F-Gal-1-P)

For detailed description, refer to Supplementary Materials and to the references [9,10,11].

### 2.3. Preparation of 2,2′-difluoro-2-deoxy-galactose (F_2_-Gal) and 2,2′-difluoro-2-deoxy-galactose-1-phosphate (F_2_-Gal-1-P) and Its per-O-acetylated Derivative

Compound 2 (Figure S6) was prepared from commercial 3,4,6-tri-*O*-acetyl-D-galactal according to the procedure reported by Suàrez et al. (27%) [12] based on Selectfluor chemistry [13,14,15]. For further methodology and structural characterization of products, refer to the Supporting Information.

### 2.4. Cells and Cell Culture

The cell model HEK293-MUC1 (previously designated MUC1-M2) has been described earlier [16]. HEK293-MUC1 and non-transfected HEK293 cells were grown at 37 °C (5% CO_2_) grown in 75 cm^2^ flasks using Dulbecco’s modified-Eagle’s medium (DMEM) with Stable glutamine/PAA (Sigma), 10% FCS, 1% PenSrep. To minimize stress reactions associated with changing the medium supplementation to either F-Gal (0.5 g/L) and 1 g/L glucose/galactose, cells were cultivated for 2 to 3 days in low-glucose medium with galactose (low- glucose 1 g/L, low-galactose 1 g/L). Afterward, the cells were finally cultivated parallel in the presence or absence of F-Gal (0.5 g/L) or F_2_-Gal for 72 h.

GALT-deficient and control fibroblasts were obtained from the NIGMS Human Genetic Cell Repository. Galactosemic fibroblasts (GM01704, male, 2 years at sampling) were homozygously affected by the mutation Gln188Arg (Q188R) in the *GALT* gene. Healthy control fibroblasts (GM05659, male, 1 year at sampling) are described as apparently healthy skin fibroblasts. Fibroblasts were cultivated parallel in 75 cm^2^ flasks to near confluence using DMEM with Stable glutamine/PAA (Sigma), 10% FCS, 1% PenStrep. Before starting the experiments, the cells were grown in low-glucose medium (glucose 1 g/L) for one day to minimize stress reactions. Finally, the healthy fibroblast was grown in the presence or absence of F-Gal or F_2_-Gal in low-Glc/low-Gal medium for 72 h. In addition, GALT-deficient cells were cultivated for 72 h in the presence of galactose and glucose (1 g/L).

### 2.5. Recombinant Expression and Purification of Human GALT in E. coli

The plasmid was generated by automated gene synthesis (General Biosystems, Morrisville, USA) and inserted into cloning vector pET-15b using cloning sites Ndel-BamH1 (insert size 1149 bp). Then, 1 μL of plasmid DNA at a concentration of approximately 200 ng/μL was used for transformation of 50 μL competent BL21(DE3)-RIPL *E. coli* cells under standard conditions. Single colonies were picked to inoculate 20 mL ampicillin LB medium, which was then incubated overnight at 37 °C, 230 rpm. Approximately, 3 mL from the starter culture was used to inoculate 1 L selection LB medium, which was incubated at 37 °C. When reaching an OD600 of approximately 0.5 isopropyl-β-D-galactopyranoside (IPTG) was added to a final concentration of 0.1–1.0 mM, and incubated at 16 °C and 230 rpm for 24 to 48 h. After induction, the *h*GALT-expressing cells were collected by centrifugation at 2890× *g* for 20 min. at 4 °C. The cell pellets were washed once with cold PBS, then resuspended in 10 mL lysis buffer (approx. 3 mL per gram wet weight). The cells were lysed on ice by sonication at 30% amplitude in 15 s intervals (15 s pulse, 60 s pause). The suspension was then centrifuged at 9.800× *g* for 30 min at 4 °C. The supernatant containing native proteins were filtered using a sterile 0.2 μm syringe filter and applied to the washed and equilibrated Ni-NTA column. The column was washed with 3 column volumes of lysis buffer, and the His-tagged protein was then eluted in 0.5 or 3 mL fractions with increasing imidazole concentrations, from 100 to 300 mM. Then, 0.5 mL Amicon Ultra 3000 MWCO centrifugal units were used as described by the manufacturer for the concentration of *h*GALT-containing fractions and buffer exchange to 0.1 M 4-(2-hydroxyethyl)-1-piperazineethanesulfonic acid (HEPES) pH 7.7. The protein concentration was determined via Bradford assay, and the concentrated *h*GALT was stored in 50% glycerol at −20 °C.

### 2.6. Kinetic Studies of Recombinant Human GALT by Coupled Enzyme Assay

The kinetic assays in the presence or absence of inhibitor (fluorinated galactose) were performed in the coupled enzyme methodology as described in [17]. Enzymatic activities of recombinant *h*GALT were determined by coupling the production of glucose-1-phosphate to its isomerization to glucose 6-phosphate and its subsequent Nicotinamide adenine dinucleotide phosphate (NADP^+^)-dependent oxidation. The reaction was performed at 25 °C and contained 10 mM HEPES, pH 8.8, 5 mM dithiotreitol (DTT), 5 mM glucose-1,6-*bis*phosphate (added as catalyst), 5 mM MgCl_2_, 0.8 mM NADP^+^, 0.03 mg glucose-6-phosphate dehydrogenase (G6PDH), and 0.4 mg of phosphoglucomutase (PGM). Neither PGM nor G6PDH were inhibited by F-Gal or F-Gal-1-P. Each assay was performed in triplicate in a 96 well plate format with total volumes of 150 μL. Kinetic constants for Gal-1P were determined at constant UDP-Glc concentration (2 mM) by varying its concentration from 0.1 to 3.0 mM. The amount of NADPH produced (detected by absorption at 340 nm) is equivalent to the amount of Glc-1P formed.

### 2.7. Analysis of Relative Conversion Rates of Gal-1-P and F-Gal-1-P

*h*GALT activity assays were performed in the presence of 0.5 mM Gal-1-P and 1.0 mM F-Gal-1-P (as a potential substrate) to generate the respective UDP-derivatives of Gal and F-Gal, which were indirectly measured via a coupled enzyme assay using N-acetylglucosamine (GlcNAc) as substrate for human β4-Gal-transferase. After 10 or 30 min, the GALT reaction was stopped by heat inactivation of the enzyme and 1 mM GlcNAc together with 1U of recombinant human β4-Galactosyltransferase (SAE0093, Sigma, Munich, Germany) were added. The reaction was stopped after overnight incubation at 37 °C and the mixture was desalted on 150 mg carbograph columns (Grace, Fisher Scientific, Schwerte, Germany) and dried by vacuum rotation before trimethylsilylation of the sugars with N-methyl-N-trimethylsilyl- trifluoroacetamide for 15 min at 70 °C. The products were analyzed by GC-MS on a 15 m RTX5-SILMS column (Restek, Bad Homburg, Germany) using a temperature gradient from 100–300 °C with 6 °C/min. Full scan spectra were recorded at 70 eV in an MD800 mass analyzer.

### 2.8. Differential N-glycosylation Analyses of Secretory Chicken Netrin-1 Formed in the Presence or Absence of F-Gal

Transfected HEK293 cells (expressing a doubly strep-tag labeled, inducible chicken netrin-1) were kindly provided by Prof. Manuel Koch (Institute of Biochemistry, Medical Faculty, University of Cologne) and cultured as described above. The medium was replaced after adherence of cells by serum-free DMEM/F-12 and cells were adapted to the low-Glc/low-Gal medium (each 0.1%) for three days before expression of netrin-1 was induced with 1 µg/mL final concentration of doxycycline. Simultaneously, the medium was inoculated with +/− 0.5 g/L of F-Gal, and cells were grown in six-well plates over 72 h before collection of culture supernatants (8 mL) with repetition for a further 72 h interval. Protein in the pooled culture supernatants was concentrated to 200 µL by ultrafiltration on Amicon-4 centrifugation devices.

To liberate N-linked chains, the glycoproteins from F-Gal-treated or control cells (approx. 350 µg) were denatured by chloroform-methanol-water extraction and after drying, the pelleted protein was digested with porcine, sequencing-grade modified trypsin (Promega, Walldorf, Germany) overnight. After heat denaturation of the protease and evaporation of the 50 mM ammonium bicarbonate buffer by vacuum rotation, the glycans were liberated in a fresh aliquot of the same buffer containing 0.5 µL of PNGaseF (Promega, 500.000 U/mL) overnight. The dried sample was taken up in 0.1% trifluoroacetic acid and glycans were separated by solid-phase extraction on C18-cartridges (100 mg, Discovery DSC-18, Supelco, Munich, Germany) before methylation. Dried samples were methylated as previously described [18]. Analysis of methylated glycans was performed on an Ultraflextreme matrix-assisted laser desorption ionization time-of-flight (MALDI-TOF-TOF) mass spectrometer (Bruker Daltonics, Bremen, Germany) in the positive ion reflectron mode using 4-hydroxy-α-cinnamic acid as matrix [18].

### 2.9. Fluorescence-Activated Cell Sorting (FACS) of HEK293 Cells and Human Fibroblasts

HEK293 and HEK293-MUC1 cells as well as the GALT-deficient and healthy fibroblasts were harvested by incubation with phosphate buffered saline (PBS) containing trypsin (0.05%), EDTA (0.02%) for 5 min at 37 °C, followed by centrifugation (5 min, 300× *g*, 4 °C). The pellets were washed 2-fold with cold PBS (4 °C) and were put on ice. Samples of 5 × 10^5^ cells were dispersed in 20 µL cold PBS and 1 µL of PE-labelled antibody (see below) or C595 antibody (anti-MUC1) was added to samples and incubated for 15 min on ice in the dark. Afterwards, 1 mL of cold PBS containing 5% FCS was added, followed by centrifugation (5 min, 400× g, 4 °C). The cells were washed twice with cold PBS/5% FCS. To stain cells for MUC1, the labeling procedure was repeated by incubation with the secondary antibody (see below). The measurements were performed on a BD FACS Canto II (BD Biosciences, San Jose, USA) using BD FACSDiva software by counting up to 10,000 cells. For data analysis, the software FlowJo (BD Biosciences) was used. The following antibodies were used: anti-CD109-PE (mouse monoclonal, Biolegend, San Diego, USA, 1:20), anti-DPP4-PE (mouse monoclonal, Biolegend, 1:20), anti-EGFR-PE (mouse monoclonal, Biolegend, 1:20), anti-MUC1 (C595, mouse monoclonal, eBioscience, ThermoFisher Scientific, Darmstadt, Germany, 1:50), secondary antibody anti-mouse-Ig-Cy5 (Jackson Immuno Research, Dianova, Hamburg, Germany, 1:50).

### 2.10. Electrophoresis and Western Blot Analysis

For a detailed description refer to Supporting Information.

### 2.11. Differential Label-Free Proteomics

Lipid raft and sample preparation: Lipid raft preparation followed previously published protocols [5]. In brief, washed pellets from about 10^7^ cells were resuspended in 1 mL PBS with protease inhibitor cocktail and 1 mL 2% Triton-X100 and incubated with rolling for 1 h at 4 °C. Lipid rafts were isolated in a sucrose gradient by increasing the sample volume to 4 mL with addition of 1.8 g sucrose (45% sucrose) and overlaying this with 4.5 and 4 mL of 35% and 5% sucrose, respectively. Ultracentrifugation was performed for 2.5 h at 198.000× *g* in an SW-41 rotor at 4 °C. The volume of a light-scattering band was taken off and lipid rafts were pelleted in a second ultracentrifugation for 1.5 h at 114.000× *g* at 4 °C. Samples for mass spectrometry were prepared after addition of 2% sodium dodecylsulfate (SDS) in PBS and transfer into 1.5 mL Eppendorf tubes by chloroform-methanol-water extraction. Protein pellets were taken up in 20 mL urea (8 M) and 4 µL protease-Inhibitor cocktail in ammonium hydrogen carbonate (5-fold). Dithiotreitol (1.2 µL, 100 mM) was added and the sample incubated for 1 h at 37 °C. Alkylation was performed by the addition of 1.8 µL 550 mM chloroacetamide (40 mM) for 30 min at RT in the dark. After predigestion with lysyl-endopeptidase (Lys-C) for 3.5 h at 37 °C, the sample was diluted with 60 µL of ammonium hydrogen carbonate (50 mM, pH 8.0) to reduce the urea concentration to below 2 M. Proteins were further digested with 0.5 µg trypsin (porcine, Promega) at 37 °C overnight. Prior to sample work-up by solid-phase extraction onto ZipTip-C18 (Merck-Millipore, Darmstadt, Germany), the peptide mixture was acidified with 10 µL of 10% formic acid.

UPLC-MS and MS/MS on a Q-Exactive Plus Orbitrap (ThermoFisher Scientific) with data processing in the Perseus framework and evaluation by bio-informatics (GO-Term Enrichment analysis, Gene Ontology Consortium) is described in detail in the Supplementary Information.

## 3. Results

### 3.1. Kinetic Studies of Recombinant hGALT Activity in the Presence of Fluorinated Galactose Inhibitors

The simulation of a galactosemic state in a cell affords a partial reduction of GALT activity by appropriate inhibitors. 2-Fluorinated galactose (F-Gal) and 2,2′-difluorinated galactose (F_2_-Gal) are expected to exert such effects, as they are taken up by the cells and both are tolerated by the galactose kinase 1 as substrates, which form the 1-phosphates, at however significantly reduced rates (Figure S1A). The formation of the respective products was verified by GC-MS identification (Figure S1B).

**Figure 1:**
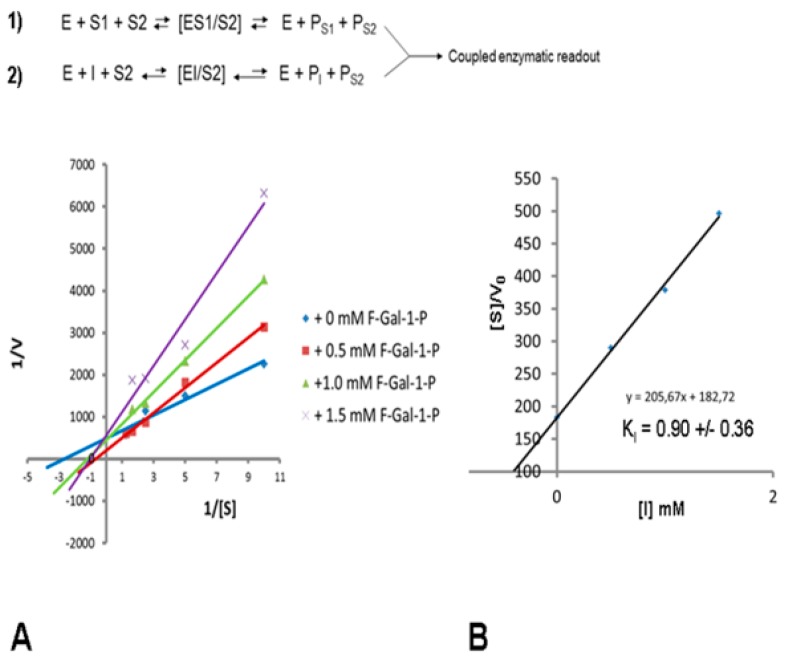
Kinetic studies of recombinantly expressed human GALT. (**A**) Lineweaver–Burk (LB) plots of two independent triplicate kinetic assays based on coupled enzyme methodology. After a pre-steady state, progress within the first 60 s linear increase of Glc-1-P formation by GALT was followed over 5 min to determine kinetic constants K_M_ and K_I_ of the substrate (Gal-1-P) or inhibitor (F-Gal-1-P), respectively. The substrate S1 (Gal-1-P) was varied from 0.1 to 0.8 mM, substrate S2 (UDP-Glc) was held constant, and inhibitor (I) was varied from 0 to 1.5 mM. The reaction scheme shown above of the LB plot refers to a partial conversion of the fluorinated inhibitor (I) to the product P_I_ (see reaction 2, which does not compete significantly with reaction 1 during the initial phase of the reaction; refer to Figure 2). (**B**) Plot of [S]/V_0_ vs. [I] to determine the K_I_ value for F-Gal-1-P.

**Figure 2:**
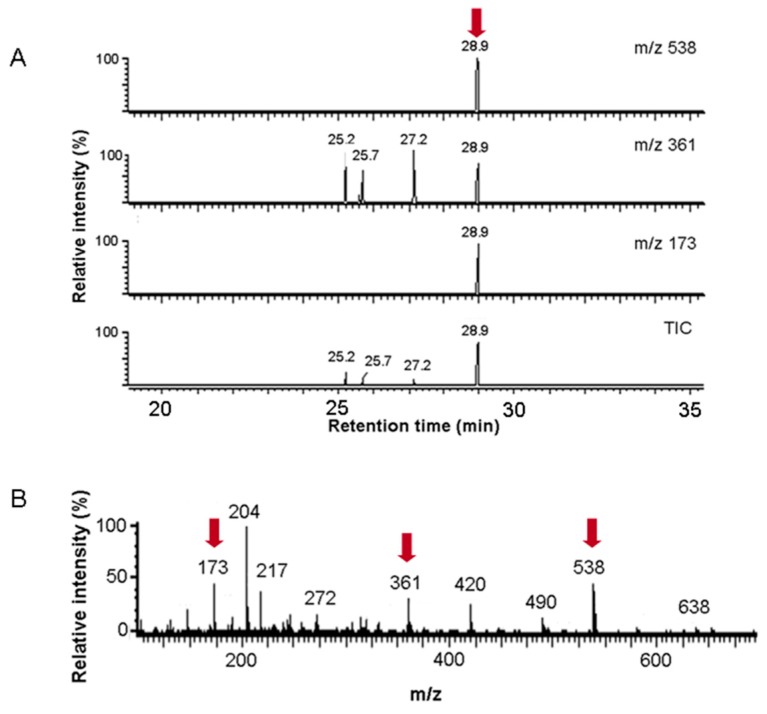
Gal-1-P and F-Gal-1-P conversion by GALT. (**A**) Total ion current (TIC) and mass selective chromatograms of trimethylsilytated oligosaccharides. The products of the GALT reaction, UDP-conjugates of either Gal or F-Gal, were indirectly detected in a coupled reaction catalyzed by recombinant human β4-galactosyltransferase converting the substrate GlcNAc into the corresponding disaccharides. The formation of products was monitored after enrichment/desalting on graphitized carbon and trimethylsilylation by gas chromatography-mass spectrometry (GCMS). Gal-GlcNAc was identified at 28.9 min (marked by red arrow) based on an authentic standard and on the electron-impact (EI) fragment spectrum (Figure 2B). F-Gal-GlcNAc at slightly decreased retention times and giving rise to alternative fragment series with incremental mass decrease of -70 amu was not detectable. (**B**) EI mass spectrum of the product at 28.9 min. The EI spectrum shows intense fragment ions (marked by red arrows) at *m*/*z* 173, 361, and 538, which characterize the trimethylsilylated disaccharide Gal1-4GlcNAc.

To confirm that 2-fluoro-2-deoxy-galactose-1-phosphate represents an inhibitor of *h*GALT, we expressed the human enzyme recombinantly in *E. coli* and measured the kinetics of the reaction at different substrate and inhibitor concentrations to determine the kinetic constants K_M_ (Michaelis constant) and K_I_ (inhibition constant). The studies were performed in a coupled enzymatic assay using glucose-1,6-phosphate mutase and glucose-6-phosphate dehydrogenase. Based on the initial velocities (time frame 1–5 min) measured at substrate and inhibitor concentrations in the range of 0.1–3 mM, we could rule out uncompetitive or noncompetitive inhibition of GALT by fluorinated galactose-1-phosphate (F-Gal-1-P). Each of these would result in either reduced or unchanged K_M_ and in reduced V_max_ values. The Lineweaver–Burk (LB) plots clearly show increased K_M_ values (Figure 1). Repetitive experiments revealed slight apparent increase of 1/v = 1/V_max_ by shifts of intersecting lines in LB plots. This effect could result from partial conversion of the inhibitor to the fluorinated product F-Gal-UDP, resulting in higher effective substrate concentration and increased Glc-1-P product formation, which is the final readout of the coupled enzymatic assay (see reaction B in Figure 1). To estimate the extent of conversion of F-Gal-1-P to F-Gal-UDP, a coupled enzyme assay using recombinant human β4-galactosyltransferase (β4Gal-T) for the conversion of GlcNAc to either LacNAc or F-LacNAc was performed. GC-MS analysis of trimethylsilylated disaccharides enriched on graphitized carbon confirmed that in a time frame up to 30 min Gal-GlcNAc, but no detectable fluorinated product F-Gal-GlcNAc was formed (Figure 2). This finding is also corroborated by the nearly unchanged amounts of fluorinated galactose-1-phosphates over 4 h. After 72 h of cell culture, we could detect considerable amounts of 2-fluoro-galactitol in the supernatants by GC-MS (Figure S2) indicating that F-Gal is partially reduced by aldolase reductase.

### 3.2. N-Glycosylation of Recombinant Probes in the Presence of Fluorinated Galactose

In reference to previous work demonstrating a dys-glycosylation induced by fluorinated galactose [19,20], we analyzed a recombinant secretory glycoprotein probe for effects on N-glycosylation in transfected HEK293 by mass spectrometry. Previous work had demonstrated a more or less complete inhibition of N-glycosylation (between 1 to 5 mM F-Gal), when cells had been grown in the presence of fluorinated galactose. To confirm these findings on a structural level, we characterized the N-glycosylation profiles of a secreted recombinant probe (inducible chicken netrin-1 expressed in HEK293 cells) generated in the presence of 2.7 mM F-Gal by applying mass spectrometry of methylated glycans. The comparative analyses of samples from F-Gal-treated vs. control cells revealed no inhibitory effect and no qualitative differences in the N-glycosylation profiles (Table 1). The profiles were dominated by bi-antennary glycans with or without core-fucosylation and mono-sialylation of one of the antennae. There was also no obvious decrease in the degree of netrin-1 glycosylation, as judged from comparative gel electrophoresis (Figure S3B).

### 3.3. Establishment of the Epithelial Cell Model (HEK293 and HEK293-MUC1)

**Figure 3:**
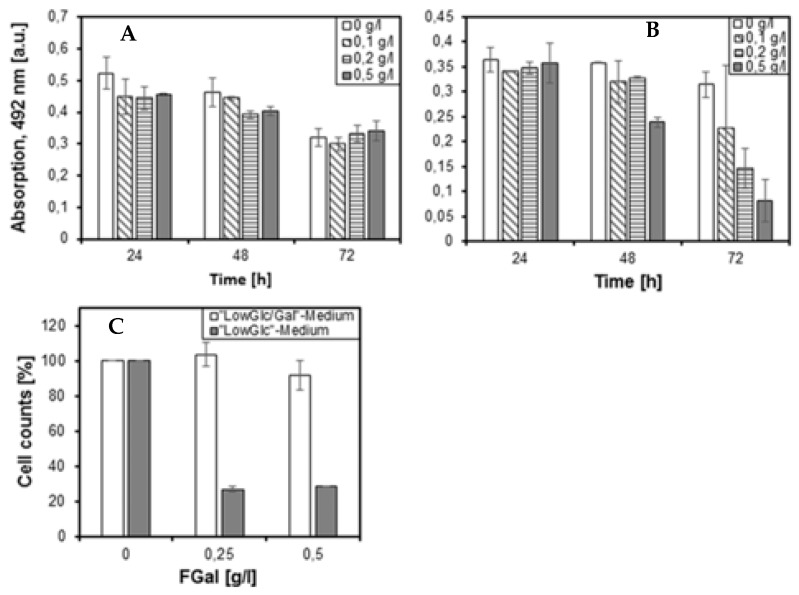
Time- and concentration-dependent viability of HEK293 cells in the presence of F-Gal. Cell viability (3-(4,5-dimethylthiazol-2-yl)-5-(3-carboxymethoxyphenyl)-2-(4-sulfophenyl)-2H-tetrazolium, MTS, staining) is shown after 24–72 h of cultivation in low-Glc/low-Gal medium (**A**) or low-Glc medium (**B**) in the absence or presence of varying concentrations of F-Gal (0–0.5 g/L). Growth rates were measured by counting cells after 72 h cultivation in either low-Glc/low-Gal or low-Glc medium in the absence or presence of inhibitor (**C**).

The adenovirus-transformed human embryonic kidney cell line HEK293 was chosen as cells have been shown to have an unexpected relationship to neurons by expressing the neurofilament subunits NF-L, NF-M, NF-H, and α-internexin as well as many other neuron-specific proteins [8]. Initial experiments were performed to exclude potential toxic effects of the fluorinated compounds on the viability of cells. Previous work had demonstrated for even low concentrations of F-Gal in rat liver cell culture (1 mM) that N-glycosylation pathways are strongly affected [19], (see also above). Under low-Glc conditions (0.1%) in the presence of the stressor galactose (0.1%, low-Gal), we could demonstrate that cells remained viable up to concentrations of 0.5 g/L of F-Gal (Figure 3A). This holds true for incubation periods of up to 72 h. However, when cells were grown in the absence of galactose, cellular growth rates were reduced by about 75% even at 0.25 g/L of F-Gal and effects on viability became apparent after 48 h in cell cultures with 0.5 g/L F-Gal (Figure 3B). Additionally, the growth rates are strongly reduced by the inhibitor in the absence of galactose (Figure 3C).

**Figure 4:**
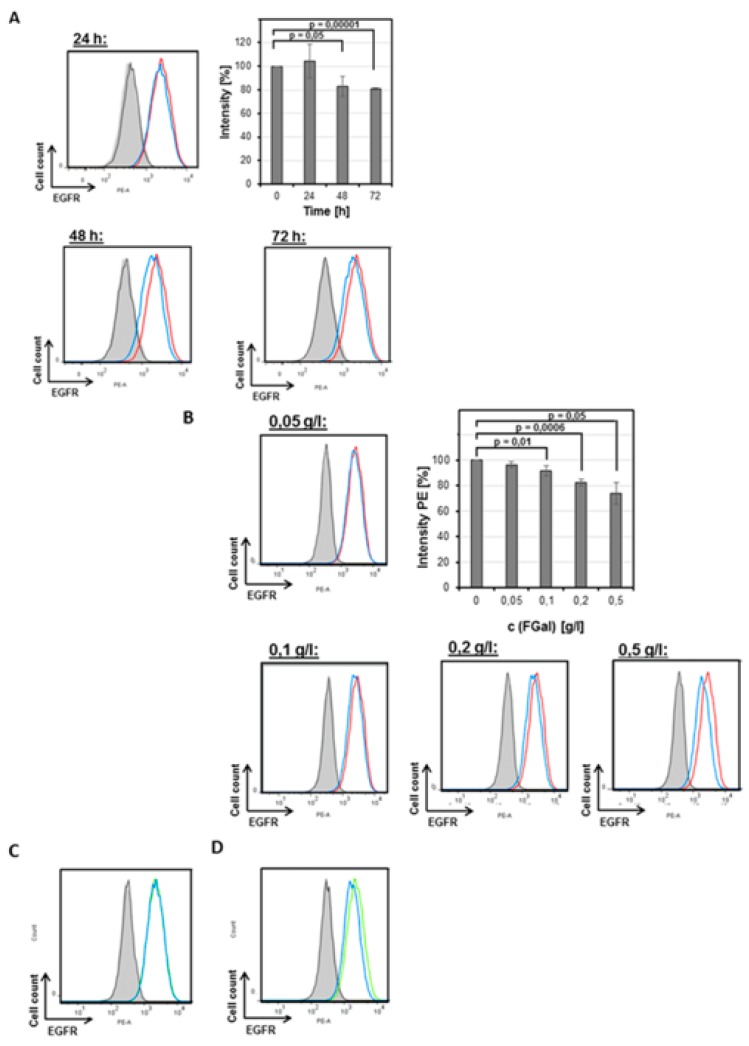
Plasma membrane marker glycoprotein EGFR shows reduced surface expression in fluorescence-activated cell sorting (FACS) induced by F-Gal in a time- and F-Gal concentration-dependent manner. Effect of varying cultivation times (**A**) and F-Gal concentrations (**B**). Control cells are shown in red, F-Gal-treated cells in blue (**A**,**B**). (**C**) Competitive effect of increased galactose concentration (4.5 g/L) in the presence of 0.5 g/L F-Gal. Control cells in low-Glc/high-Gal medium without F-Gal are shown in green, cells treated with F-Gal in blue. (**D**) Rescue effect by increased F-Gal concentration (1 g/L) in the presence of 4.5 g/L galactose. Control cells without F-Gal in low-Glc/high-Gal medium are shown in green, cells treated with F-Gal in blue.

To establish parameters for effective GALT inhibition and associated changes of protein expression in the plasma membrane, we chose EGFR as a readout, which had been described to show decreased expression on galactosemic fibroblasts [6]. When grown under low-Glc/low-Gal conditions, the marker protein did not show any reduced expression up to 24 h, however, on longer incubation times, a significant decrease in surface expression became obvious in FACS analyses (Figure 4A). This effect was dependent on the concentration of F-Gal (Figure 4B).

Keeping the F-Gal concentration at 0.5 g/L (2.77 mM) over 72 h at varying galactose concentrations, revealed the expected competitive effects of the non-fluorinated and fluorinated compounds. No obvious effects on EGFR surface expression were observed in the absence of F-Gal, when comparing cells grown under low-Glc/low-Gal and low-Glc/high-Gal conditions. However, the reduced surface expression of EGFR observed in the presence of 0.5 g/L F-Gal under low-Gal conditions was rescued by higher galactose concentrations (Figure 4C). Accordingly, under high-Gal conditions, the reduced surface expression phenotype of HEK293 cells could only be induced by higher F-Gal concentrations (Figure 4D).

### 3.4. FACS Reveals Altered Plasma Membrane Localisation of Glycoprotein Markers Induced by F-Gal and F_2_-Gal

**Figure 5:**
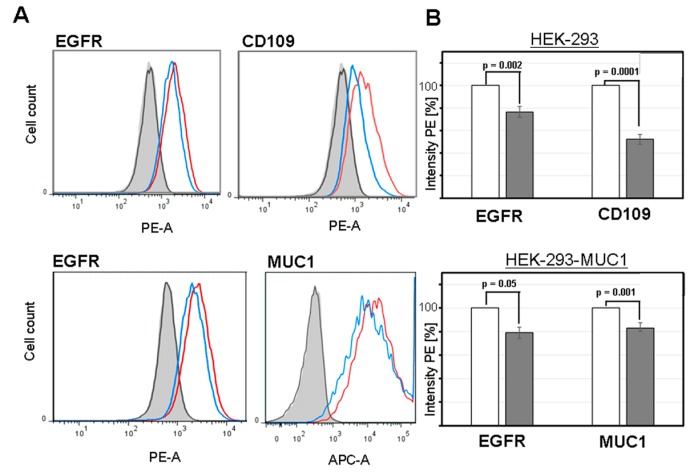
Plasma membrane marker glycoproteins EGFR, CD109, and MUC1 show a decrease of surface expression in fluorescence-activated cell sorting (FACS) induced by F-Gal. (**A**) Cell separation by triplicate FACS analyses revealed changes of membrane marker expression in HEK293 and HEK293-MUC1 cells in the absence (red) or presence of F-Gal (blue). Cells were cultured in low-Glc/low-Gal medium. (**B**) The bar diagram shows relative fluorescence intensities (%) for HEK293 or HEK293-MUC1 cells grown in the absence (white) or presence of F-Gal (dark grey). Cells were cultured in low-Glc/low-Gal medium.

Differential proteomics of galactosemic fibroblasts had revealed a series of membrane-bound glycoprotein receptors that showed concerted decreases in their surface expression in lipid rafts [5]. Besides EGFR, the surface expression of markers CD109, DPP4, and GPNMB were decreased in patient-derived fibroblasts and were postulated to show similar reduced expression in our cell model. As a typical epithelial cell surface marker, we used MUC1 in a MUC1-transfected HEK293 cell line [16]. HEK293 and HEK293-MUC1 cells expressed CD109, but neither DPP4 nor GPNMB were detectable at their surfaces. HEK293-MUC1 cells were positive for the mucin (Figure 5A). The two expressed marker proteins, CD109 and MUC1, showed the expected decrease in surface exposure, when HEK293 cells were grown in the presence of F-Gal inhibitor. The triplicate assays revealed a reduction of more than 20% (EGFR) and 50% (CD109), respectively (Figure 5B). About 20% reduction of MUC1 surface expression was observed in the HEK293-MUC1 cell model. CD109 and MUC1 specific antibody binding to the cells showed strong heterogeneity in marker expression, which might be explained by glycosylation-dependent epitope access by the antibody. This is particularly true for the tandem repeat peptide epitope of MUC1, which is known to be masked by dense O-glycosylation.

**Figure 6:**
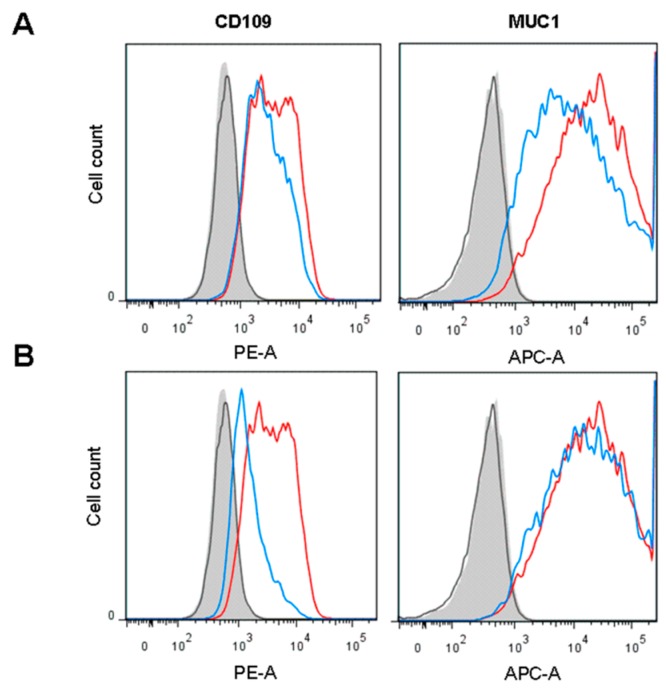
CD109 and MUC1 surface expression decrease in fluorescence-activated cell sorting (FACS) induced by F_2_-Gal and acetylated F_2_-Gal-1-P. (**A**) FACS separation diagram of HEK293-MUC1 cells grown in the absence (red) or presence of F_2_-Gal (blue). Cells were cultured in low-Glc/low-Gal medium. (**B**) FACS diagrams of HEK293-MUC1 cells grown in the absence (red) or presence of acetylated F_2_-Gal-1-P (blue). Cells were cultured in low-Glc/low-Gal medium.

As specific activity of GALT decreased to similar levels in the presence of doubly fluorinated F_2_-Gal inhibitor, we expected to see also comparable effects of this compound on surface expression of CD109 and MUC1. For the highly N-glycosylated CD109, its surface reduction was largely confined, however, to the cellular subpopulations with higher antigen expression. On the other hand, the heavily O-glycosylated MUC1 showed a significant reduction in surface expression over the entire range of cellular populations with higher or lower antigen expression (Figure 6A). Assuming that the observed effects on glycoprotein surface expression should depend in part on the rapid formation of inhibitory F_2_-Gal-1-P, we incubated HEK293-MUC1 cells in the presence of acetylated F_2_-Gal-1-P, which crosses membranes independent of facilitated diffusion transport by the cells and should mediate strong inhibitory effects after enzymatic de-O-acetylation. As expected, a moderately increased attenuation of CD109 surface expression was measurable by FACS analysis (Figure 6B). The still moderate effect on surface expression of membrane proteins (in particular seen for MUC1) could result from slow turnover rates, as is evident from EGFR and MUC1 Western blots of cell lysates (Figure S3A). Neither EGFR nor MUC1 showed decreased molecular masses after 72 h incubation in the presence of F-Gal or F_2_-Gal. A decrease in molecular masses had previously been demonstrated for F-Gal treated rat liver cells, which expressed a series of de novo synthesized radiolabeled glycoproteins with decreased glycosylation and molecular masses [19].

**Figure 7:**
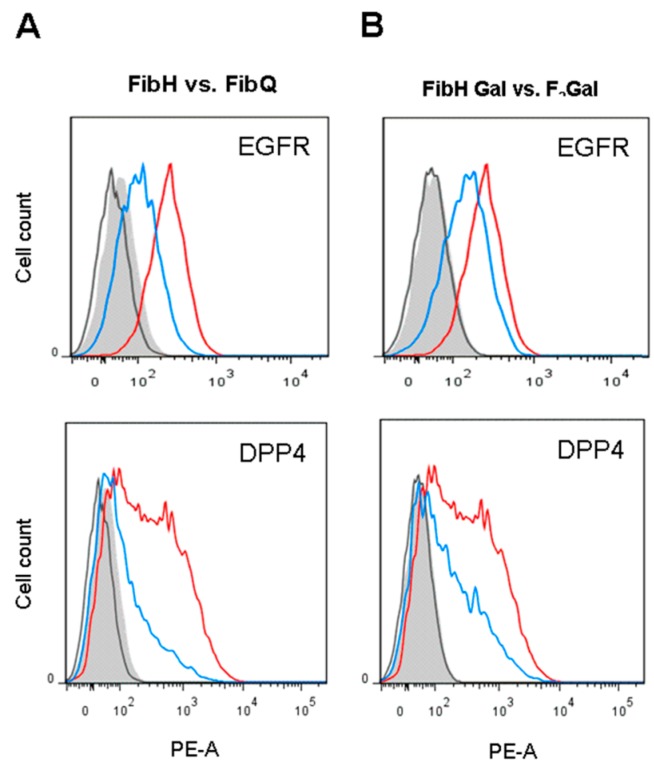
Fluorescence-activated cell sorting (FACS) experiments with galactosemic (FibQ, GM01704) and healthy control fibroblasts (FibH, GM05659) in the absence or presence of F_2_-Gal (1 g/L). (**A**) surface expression of EGFR and DPP4 on galactosemic (blue) and healthy control fibroblasts (red) in low-Glc/low-Gal medium; (**B**) surface expression of EGFR and DPP4 on healthy control fibroblasts in the absence (red) and presence of 0.1% F_2_-Gal (blue) in low-Glc/low-Gal medium.

The previously studied fibroblast model (Staubach et al. 2017) was analyzed as a reference under the same conditions. Fibroblasts from patients suffering from classic galactosemia (GM01704, Q188R) and normal control cells (GM05659), both grown under low-Glc/low-Gal conditions, were analyzed by comparative FACS and showed the expected effects on surface expression for two selected marker proteins (Figure 7A). Based on these findings, we further tried to induce the galactosemic phenotype in normal fibroblasts by cultivating these cells in the presence of F_2_-Gal. Using 1 g/L of F_2_-Gal, significant effects on surface expression of EGFR and DPP4 were measurable for inhibitor-treated normal fibroblasts (Figure 7B). In conclusion, FACS analysis demonstrated changes in membrane glycoprotein expression in MIG-HEK293 cells and fibroblasts. Moreover, the experiments with HEK293 cells and fibroblasts from healthy donors confirmed that normal cells can be made “galactosemic” by treatment with fluorinated galactoses.

### 3.5. Differential Proteomics of Lipid Rafts from MIG-HEK293 and Control Cells

A previous differential proteomics study of sex-matched normal and galactosemic fibroblasts had revealed the affection of several pathways or biological processes when cells were grown in the presence of the stressor galactose (low-Glc/low-Gal conditions) [5]. A series of galactose-induced effects were observed, i.e., an overexpression of endoplasmic reticulum (ER)-stress markers, like the chaperone GRP78 and calreticulin, which is involved in N-glycoprotein quality control. Moreover, the membrane-anchored receptor CD109 was decreased together with cadherin-13, GLIPR1, glypican-1, and semaphorin-7A [5]. To compare these findings in a fibroblast model with cellular effects induced metabolically in epithelial cells, we performed a similar study on lipid raft proteomes in the MIG-HEK293 cell model (Table S1, Figures S4–S6).

**Figure 8:**
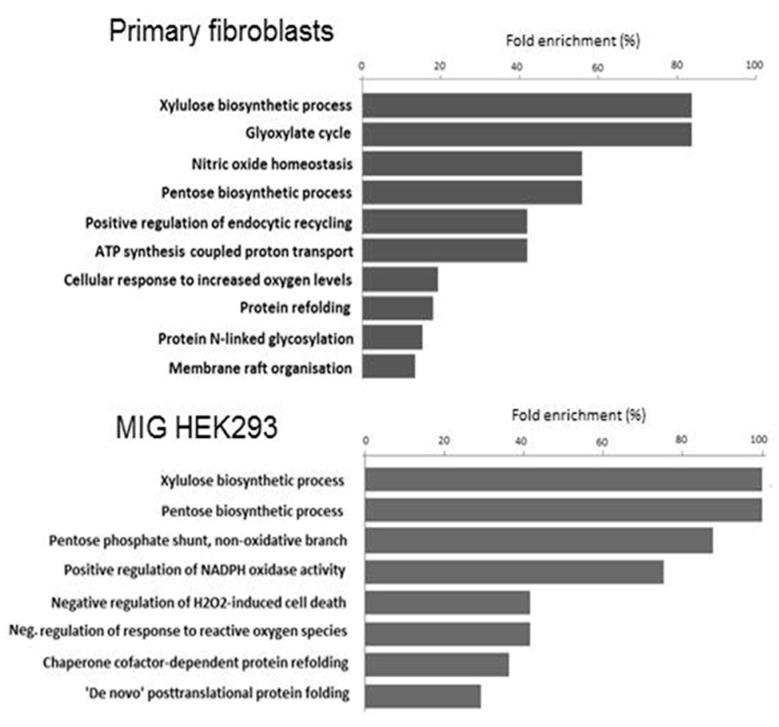
Comparative Gene Ontology Term Enrichment (GOTE) analyses of increased, fold-changed proteins in response to galactose in the MIG (metabolic induction of glactosemia) HEK293 cell model and in the galactosemic fibroblast model.

Among more than 1718 totally identified proteins, typical lipid raft proteins, like caveolin-1, flotillin-1 or -2, annexins, and cadherins were found together with a series of raft-associated proteins, like HSP90 (Table S1A). Of the quantified proteins, 76 species showed significant increase in abundance (difference of log-*p* values > 1), whereas 208 proteins were decreased in abundance (difference of log-*p* values < −1) (Table S1B). Top-ranking terms in a Gene Ontology Term Enrichment (GOTE) analysis were related to “negative regulation of intrinsic apoptotic signaling pathway in response to hydrogen peroxide”, and “negative regulation of hydrogen peroxide-mediated programmed cell death” for proteins with increased abundance (Figure 8, Table S2). For proteins with decreased abundances, the term related to “response to rapamycin” was top-ranking (Table S3), which points to the affection of the TORC1 (target of rapamycin complex 1) formation, a potent anabolic regulator of cellular growth and metabolism, that in its active state, has a lysosomal localization and is released from the Rag GTPases into the cytoplasm on amino acid starvation.

A GOTE analysis of affected biological processes revealed also other pathways, like “chaperon cofactor-dependent protein folding” (for increased species), and “protein targeting to ER”, “cotranslational protein targeting to membrane”, “protein localization to ER”, and “protein targeting to membrane” (for decreased species) among the top 30 listed according to “fold enrichment”. Among proteins with increased abundance, calreticulin and endoplasmin were listed, both involved in protein folding and quality control of glycoproteins in the ER (Figure 8).

### 3.6. Differential Proteomics of Lipid Rafts from Galactosemic and Healthy Control Fibroblasts

To compare data from differential proteomics of the galactosemic fibroblasts model with those of the MIG-HEK293 cell model (see above), we re-analyzed the galactosemic fibroblast line GM01704 (Q188R) and healthy control fibroblasts (GM05659) for differential protein expression in lipid rafts. Of the total 917 proteins identified in this experiment, 448 were quantified and showed decreased (98) or increased abundances (242) in rafts from galactosemic fibroblasts (Table S4). Strong overlap of the fibroblast cell model with the MIG HEK293 cell model was revealed (Figure 8), as the most high-ranking terms in the GOTE analysis referred also to increased abundances of proteins involved in the pentosephosphate cycle (Table S5). Additionally, proteins involved in a response to oxidative stress were strongly enriched. In good agreement with previously obtained data, the “unfolded protein response” and a number of terms related to targeted protein transport showed up in the list of affected biological processes. Besides these obvious overlaps (Figure 8), the two cell models (F-Gal treated HEK293 cells vs. galactosemic fibroblasts) revealed a series of distinct cellular pathways that were affected under galactose stress. A strong enrichment of proteins related the “protein N-glycosylation” pathways and to a “positive regulation of endocytic recycling” was revealed for galactosemic fibroblasts (Table S5). Among the top-ranking GO terms, the “glyoxylate cycle” is listed. This term actually refers to the two NADP-specific isocitrate dehydrogenases, IDH1 and IDH2. The respective proteins in the cytosol and mitochondria regenerate NADPH from NADP+ by oxidation of isocitrate. Among proteins strongly decreased under galactose stress, BASP1 and NEGR1 should be mentioned (Table S4). While the former is functionally connected with gonad, kidney, and brain development, the latter is associated with GO terms related to “locomotory behavior” and “positive regulation of neuron projection development” (uniprot). Proteins decreased in lipid rafts from galactosemic fibroblasts that are related to “axon extension” were found 18-fold enriched in the GOTE analysis (Table S6).

## 4. Discussion

### 4.1. General Discussion

The metabolic induction of a galactosemic phenotype in cells offers a variety of advantages, as it allows to study any arbitrary line for potential effects on the membrane raft proteomes and on cellular pathways that could be disease-related and give hints to organ- and tissue-specific molecular pathomechanisms. Of course, there are alternative strategies that could be followed, like the gene knockout by CRISPR/Cas9 or the site-directed mutagenesis of R188 to Gln in the *GALT* gene, which can be applied to generate either cells lacking any GALT enzyme activity or cells with a similarly reduced activity as found in cells from galactosemic patients. This residual enzyme activity is also mimicked in MIG cells, however the level of activity remaining is significantly higher and could resemble the phenotypes of “Duarte” variants, which can express between 25% and 75% of normal GALT activity.

Mammalian and non-mammalian animal models for classic galactosemia have been described [21,22,23]. However, insight into disease-associated pathways is still limited. In a yeast model, the unfolded protein response was found to have a protective role in *GALT7*-deficient cells [21]. In a *Drosophila melanogaster* model of classic galactosemia [22], a series of experimental approaches showed an increased vulnerability of GALT-deficient flies to locomotor complications. In line with these findings, a new recently constructed mouse model of the disease revealed abnormal changes in the Purkinje and the outer granular cell layers of the cerebellum in galactose-intoxicated mice [23].

### 4.2. HEK-293 Cells Grown in the Presence of Fluorinated Galactose Show a Metabolically Induced Galactosemic Phenotype (MIG)

**Figure 9:**
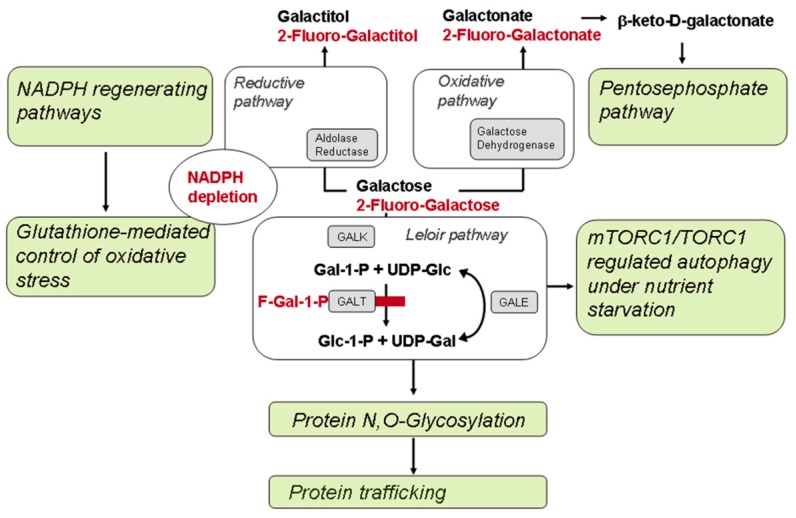
Affected pathways in epithelial MIG cell model (HEK293) of classic galactosemia according to differential proteomics. Galactose can enter the Leloir pathway with formation of Gal-1-P, but is converted at reduced rates to the UDP-Gal due to a competitive inhibition by fluorinated galactose-1-phosphate. Pathways are affected that are related to protein folding, quality control, glycosylation, and targeting to the plasma membrane (contrasting with unaltered N-glycosylation of inducible netrin-1, see above). On the other hand, fluorinated galactose apparently contributes to a depletion of the nicotinamid adenine diphosphate hydrogen (NADPH) pool by aldolase reductase inducing an up-regulation of enzymes involved in the regeneration of NADPH. There is also an impairment of the energy metabolism that could be related to phosphate entrapment by Gal-1-P and F-Gal-1-P. F-Gal-1-P in the figure corresponds to 2-fluoro-2-deoxy-galactose-1-phosphate.

MIG cells simulate the galactosemic phenotype of GALT deficient or activity-reduced cells in several respects: 1) Treated cells exhibit reduced surface expression of various glycosylated receptors. 2) Moreover, these MIG cells show affection of pathways related to energy metabolism; 3) of pathways related to the unfolded protein response; and 4) pathways related to protein trafficking and cellular localization. The role of galactose in this system is ambivalent, as it is essential as a metabolic stressor on the one side, but on the other, it competes with the fluorinated metabolites that block GALT. Based on kinetic studies with recombinant human GALT, there is a competitive effect of F-Gal-1-P vs. Gal-1-P for substrate binding sites on the enzyme. However, cells may also respond to fluorinated galactose in the same way as to accumulating galactose when trying to eliminate the stressor via reductive or oxidative pathways that surpass the Leloir pathway. In this way, fluorinated galactitol can be formed by the aldose reductase with concomitant depletion of the NADPH pool (Figure 9). Obviously, in HEK293 cells, the latter pathways play more prominent roles than the conversion of F-Gal to the UDP-activated intermediates that may ultimately lead to the formation of Dol-PP-derivatives of F-Gal and to a block of N-glycosylation [20].

We were not able to demonstrate effects on N-glycosylation of doxycyclin-inducible netrin-1 expressed in HEK293 cells in the presence of fluorinated galactose. These results obtained by mass spectrometric N-glycan profiling are in striking contrast to previous reports of two groups that were claiming a more or less complete abrogation of N-glycosylation in the presence of fluorinated galactose above 1 mM [19,20]. However, it has to be pointed out that these effects may be cell-type restricted and cannot be expected to play a major role in HEK293 cells. These show a very slow conversion of F-Gal-1-P to the UDP-activated product, as revealed in coupled enzyme assays. In other cell types, the UDP-derivative of F-Gal could be converted by UDP-galactose 4-epimerase (GALE) to fluorinated UDP-Glc that may ultimately form dolichol-P-2-deoxy-2-fluoro-glucose exhibiting inhibitory effects on the assembly and Asn-transfer of glycans to proteins. Ultimately, the lack of effects on N-glycosylation by F-Gal supports the hypothesis that a reduced surface expression of N-glycoproteins should not be related to a dys-glycosylation.

Reduced surface expression of N-glycosylated receptors, like EGFR or CD109, could result from decreased overall expression rates in cells under Gal/F-Gal stress. To discriminate the latter from a glycosylation- and transport-dependent decrease in surface exposure, we compared differential proteomic data obtained from lipid raft fractions with those from total HEK-293 cells (Table S7). However, N-glycoproteins with plasma membranous topology or in total cell proteomes did not show sufficient overlap. For this reason, we cannot support by experimental data that the reduced surface expression of selected glycosylated marker proteins is linked to a malfunction of targeted transport to the plasma membrane. Interestingly, a selected number of N-glycoproteins with plasma membranous expression showed no decrease of their abundancies in total cell proteomes of F-Gal-treated vs. untreated HEK-293 cells.

### 4.3. Molecular Pathways Affected in Classical Galactosemia

A major finding of the current study was revealed by differential proteomics of F-Gal-treated HEK293 cells, as these cells showed a preferential involvement of biological processes related to sugar metabolism (Figure 9). Top ranking biological pathways according to GO Term Enrichment (GOTE) analyses were “negative regulation of intrinsic apoptotic signaling pathway in response to hydrogen peroxide”, and “negative regulation of hydrogen peroxide-mediated programmed cell death” (Table S2). These GO terms point indirectly to a depletion of the NADPH pool and to its regeneration by glucose-6-phosphate dehydrogenase or by NADP-specific isocitrate dehydrogenases IDH1 or IDH2 (Figure 9). Besides the Leloir pathway, galactose can be metabolized via reductive and oxidative pathways [24]. The conversion of accumulating galactose in GALT-deficient cells into galactitol depletes these of NADPH, which is the cosubstrate of glutathion reductase, and hence indirectly involved in the scavenging of hydrogen peroxide-derived free radicals and the reduction of oxidative stress. The galactosemic phenotype could hence be associated with an up-regulation of NADPH generating pathways and concomitantly with a negative effect on hydrogen peroxide-mediated programmed cell death. The non-oxidative part of the pentosephosphate shunt is also involved in the conversion of another possible metabolite of accumulating galactose, the oxidatively generated galactonate, which is either excreted or converted to β-keto-D-galactonate and decarboxylated to xylulose (Figure 9). NADPH is partially generated by glucose-6-phosphate dehydrogenase (G6PDH) in the pentosephosphate cycle, however, cytosolic malate dehydrogenase (MDH1) and isocitrate dehydrogenase IDH1 contribute also significantly to the NADPH pool. The latter enzyme together with its mitochondrial isoform IDH2 were found in our study with increased abundances in galactosemic fibroblasts, whereas MDH1 was increased in the MIG model of HEK293 cells.

Among proteins with decreased abundance, those show strongest enrichment that are related to the biological processes “amino acid starvation” and “response to rapamycin” (Table S3). These GO terms indicate affection of mTOC1/TORC1 regulated autophagy occurring under nutrient starvation conditions [25,26,27] (Figure 9). LAMTOR3, which lists among proteins with increased abundance, is part of the Ragulator complex and known to function in amino acid sensing and the activation of mTORC1. The target of rapamycin complex is involved in signaling that promotes cell growth in response to growth factors, energy levels, and amino acids. In this context, the accumulation of galactose-1-phosphate in patients with classic galactosemia has been connected with phosphate trapping and effects on energy metabolism. Galactose-1-phosphate is also known to inhibit glycogen phosphorylase, which results in hypoglycemia and again affects the energy metabolism of galactosemic cells. In a previous study on galactosemic fibroblasts, changes in protein expression indicated a glucose deprivation under galactose stress, which resulted in a concerted decrease of enzymes involved in electron transport, increased mitochondrial ATPases, and lysosomal LAMTOR4 [25]. In the current re-investigation of lipid raft proteins in a fibroblast model, we found LAMTOR1 strongly increased (Table S4).

## 5. Conclusions

In conclusion, from the above presented and discussed results, we claim that the approach to metabolically induce a galactosemia-like phenotype in healthy epithelial cells is feasible and should be transferable to other cell lines, like neuroblastoma cells. We expect that future studies on these stem cell-like neuronal cells will allow to elucidate more specifically those molecular pathomechanisms associated with developmental changes in the brain of patients suffering from classic galactosemia. One of the major findings of the current study refers to the identification of concertedly affected pathways in galactosemic cells and MIG-HEK293 cells, which both showed a strong response to oxidative stress, a phenomenon that was also revealed in the *Drosophila* model of classic galactosemia and represents a common feature of various other diseases [28].

## Figures and Tables

**Table 1:** Mass spectrometric N-Glycoprofiling of chicken netrin-1 expressed in HEK293 cells in the absence or presence of F-Gal.

Molecular Mass	Glycan Composition	Structure Model	Presence in Sample	
MNa/MH-32			Control	F-Gal
1580/1526	H5N2	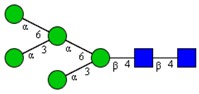	++	++
1866/1812	H4N4	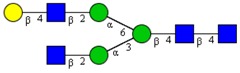	+	+
2040/1986	F1H4N4	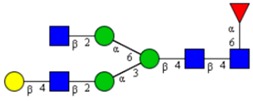	+	+
2070/2016	H5N4	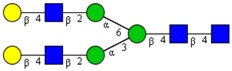	++	++
2244/2190	F1H5N4	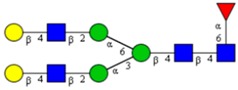	+++	+++
2285/2231	F1H4N5	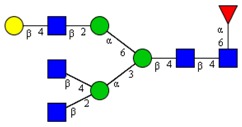	++	+++
2431/2377	S1H5N4	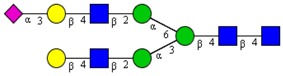	+++	++
2605/2551	S1F1H5N4	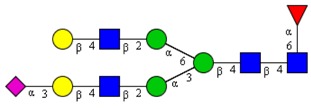	++	++
2967/2913	S2F1H5N4	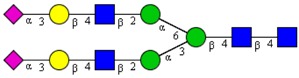	(+)	(+)
3055/3001	S1F1H6N5	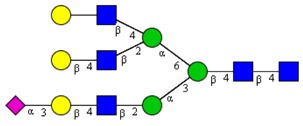	(+)	(+)

Semi-quantitative evaluation was based on peak intensities relative to base peak intensities in matrix-assisted laser desorption ionization mass spectrometry (MALDI-MS) survey spectra (mass given in boldface): (+) below 10% of base peak, + below 30% of base peak, ++ above 30% of base peak, +++ 90–100% of base peak. Structure models refer to representative examples of possible isomers. Detected molecular ions are underlined.

## Supplementary Materials

The following are available online at https://www.mdpi.com/2073-4409/9/3/607/s1. Figure S1. Relative activities of galactokinase-1 in phosphorylation of Gal, F-Gal, and F_2_-Gal (A), and EI spectra of a GC-MS analysis of GalK1 products (B); Figure S2. GC-MS analysis of F-galactitol in HEK293 culture supernatants. Figure S3. Western blots of EGFR and MUC1 in HEK293-MUC1 cell lysates. Figure S4. Volcano plot of –log p-values vs. t-test difference. Figure S5. Scatter plots indicating the calculated correlation between the datasets. Figure S6. Heatmap showing relative expression levels of the proteins according to a clustering behavior. Figure S7. Syntheses of 2,2′-difluoro-galactosides F_2_-Gal and F_2_-Gal-1P. Table S1. List of total and fold-changed proteins with confident level identification referring to the HEK293 cell model. Table S2. GO Term Enrichment Analysis (Biological Process) of significantly increased proteins (HEK293 cell model). Table S3. GO Term Enrichment Analysis (Biological Process) of significantly decreased proteins (HEK293 cell model). Table S4. List of quantified proteins identified in galactosemic and control fibroblast lipid rafts. Table S5. GO Term Enrichment Analysis (Biological Process) of significantly increased proteins (fibroblast model). Table S6. GO Term Enrichment Analysis (Biological Process) of significantly decreased proteins (fibroblast model). Table S7. Total cell protein expression of HEK-293 cells in the presence or absence of F-Gal.
